# Study of Ionization Charge Density-Induced Gain Suppression in LGADs

**DOI:** 10.3390/s22031080

**Published:** 2022-01-30

**Authors:** M. Carmen Jiménez-Ramos, Javier García López, Adrián García Osuna, Iván Vila, Esteban Currás, Richard Jaramillo, Salvador Hidalgo, Giulio Pellegrini

**Affiliations:** 1 Centro Nacional de Aceleradores (CNA), 41092 Sevilla, Spain; fjgl@us.es (J.G.L.); agosuna@us.es (A.G.O.); 2 Departamento de Física Aplicada II, Universidad de Sevilla, 41012 Sevilla, Spain; 3 Departamento de Física Atómica, Molecular y Nuclear, Universidad de Sevilla, E-41080 Sevilla, Spain; 4 Instituto de Física de Cantabria (IFCA-UC-CSIC), 39005 Cantabria, Spain; ivan.vila@csic.es (I.V.); jaramilo@ifca.unican.es (R.J.); 5 Solid State Detector Group, CERN, CH-1211 Genéve 23, Switzerland; esteban.curras.rivera@cern.ch; 6 Instituto de Microelectrónica de Barcelona (IMB-CNM, CSIC), Universitat Autònoma de Barcelona, 08193 Barcelona, Spain; salvador.hidalgo@csic.es (S.H.); giulio.pellegrini@csic.es (G.P.)

**Keywords:** LGAD, IBIC, TRIBIC, gain suppression, microplasma generation, Bragg peak

## Abstract

Gain suppression induced by excess carriers in Low Gain Avalanche Detectors (LGADs) has been investigated using 3 MeV protons in a nuclear microprobe. In order to modify the ionization density inside the detector, Ion Beam Induced Current (IBIC) measurements were performed at different proton beam incidence angles between 0° and 85°. The experimental results have been analyzed as a function of the ionization density projected on the multiplication layer, finding that the increase of ionization density leads to greater gain suppression. For bias voltages close to the gain onset value, this decrease in gain results into a significant distortion of the transient current waveforms measured by the Time-Resolved IBIC (TRIBIC) technique due to a deficit in the secondary holes component. For angles of incidence such that the Bragg peak falls within the sensitive volume of the detector, the formation of microplasmas modifies the behavior of the gain curves, producing an abrupt decrease in gain as the angle increases.

## 1. Introduction

Low Gain Avalanche Detectors (LGADs) are *n*^+^-on-*p* silicon sensors with intrinsic gain [1]. This technology has been developed in the framework of the RD50 Collaboration [2] and is based on the standard Avalanche Photo Diodes (APD). The internal gain is achieved by implantation of a *p*^+^ multiplication layer between the *n*^+^ contact and the *p* substrate. When the detector is biased, in the multiplication layer, a very strong electric field is created which induces the avalanche multiplication—impact ionization—of the electrons passing through it, thus creating additional electron-hole pairs. The gain values presented by LGADs are moderate (10–50), without breakdown, and increase smoothly with the applied voltage when the sensor is reverse biased (working in linear mode before breakdown). These gains need not be as high as those of APD detectors (usually with gain values >100, working in Geiger mode after breakdown) because, for measuring high-energy charged particles, it is not a requirement to have such high signal amplifications as for measuring low energy signals, which is a typical application of APD detectors. 

The use of LGADs is foreseen in the Large Hadron Collider (LHC) upgrade in cases where, in addition to good spatial resolution, excellent temporal resolution is needed to correctly determine and assign traces where there are a large number of individual interaction vertices (pile-up). In the High Luminosity upgrade of the European Laboratory for Particle Physics (CERN—HL-LHC), the pile-up factor is expected to be four times higher than in the current LHC experiments [3]. Therefore, both ATLAS and CMS plan to introduce sub-detectors in order to perform timing measurements of Minimum Ionizing Particles (MIPs) [4,5] that will require timing capabilities of the order of ~30 ps. To this end, thin LGADs for the High Granularity Time Detector (HGTD) have been proposed as an option in ATLAS and, in CMS, this technology has been proposed for the MIP timing detector (MTD) [6,7].

**Figure 1:**
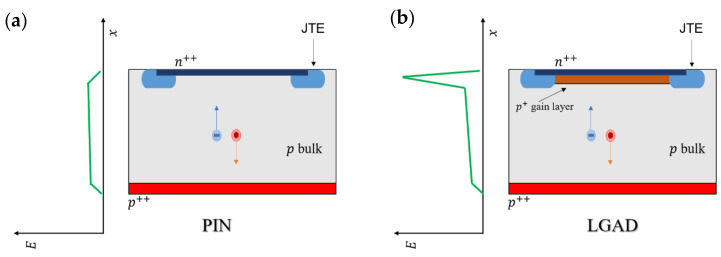
(**a**) a schematic of the cross-section of a pad-like PIN detector and a qualitative profile of the electric field amplitude is shown. (**b**) a schematic of the cross-section of a pad-like LGAD and qualitative profile of the electric field amplitude is shown, there is a peak located in the same region of the gain layer in which the avalanche happens.

Schematics of the cross-section of a standard LGAD and a twin PIN diode, along with the electric field profile in each case can be seen in Figure 1.

To ensure a good interpretation of the data obtained in an experiment with LGADs, it is essential to know the gain value at all times, as gain variations during sensor operation can degrade the temporal resolution [8,9]. The gain is dependent on temperature and reverse bias voltage, and also changes when the detector is damaged if it is subjected to a high radiation field [9]. This is why it is necessary to keep the applied voltage and temperature well controlled during an experiment and to know how the gain versus voltage curves change as the detector is irradiated, i.e., when the fluence to which it is exposed increases. In this way, gain changes can be corrected by raising the applied voltage.

Another effect that can induce gain suppression in LGADs is the formation of microplasmas in the bulk due to the generation of a high ionization density, i.e., a high carrier density along the particle’s track [10,11]. However, when the ionization trace is below the regime of microplasma formation, a decrease in detector gain was not expected. Contrary to this assumption, a recent study has shown that even the ionization density produced by a MIP when passing through an LGAD generates gain suppression, and this is because the electric field suffers a drop when the generated carriers reach the multiplication layer [12]. In that work, it is shown how the gain measured with an infrared (IR) laser and a Sr-90 beta emitter differs at a given voltage. Although the IR-laser intensity was adjusted to generate the charge equivalent to that produced by a MIP (the electrons from the Sr-90 source), the ionization density generated by both probes is different due to the difference in the volume of the generated track, i.e., in the case of the Sr-90, the charge is generated in a much narrower ionizing path so the projection of the ionization density onto the multiplication layer is much larger. When a large density of carriers reaches the multiplication layer, there is a local drop in the electric field which causes the impact ionization parameter to decrease, resulting in a lower gain [13]. That research has shown that the comparison between gain curves obtained using different types of ionization sources is not adequate, since the gain does not depend only on the charge generated by the primary beam, but also on the distribution of the ionization density generated, even when no microplasmas are generated. This makes knowing the ionization density when using LGADs a fundamental factor when interpreting the results obtained, as well as knowing the temperature, the applied voltage and the fluence at which they have been tested.

**Figure 2:**
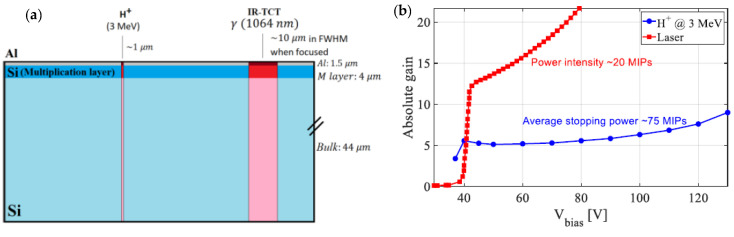
(**a**) LGAD structure and details of IBIC and TCT measurements (Al: aluminum layer; M: multiplication layer; figure not to scale). (**b**) Absolute gain curves obtained by IBIC and TCT with IR-laser.

In this work, Ion Beam Induced Charge (IBIC) and Time Resolved-IBIC (TRIBIC) measurements have been performed with 3 MeV protons to characterize a LGAD detector. The absolute gain curves obtained by IBIC and the one measured by Transient-Current Technique (TCT) with an IR-laser of equivalent intensity to approximately 20 MIPs differ significantly (Figure 2), implying that the gain suppression when using 3 MeV protons is important. Note that the protons used generate a charge equivalent to that of 75 MIPs and the trace volume is about 10 times smaller compared to a focused IR-laser (Figure 2), so in this case the ionization densities differ by about a factor 40.

The objective of this work is to perform a detailed study of the dependence of the absolute gain on the projected ionization density in the gain layer (from now on, this quantity will be referred to as the linear ionisation density, λ). For this purpose, IBIC measurements were performed at different angles of incidence on twin PIN and LGAD detectors, differing only in the implantation of the gain layer in the later. The angular study of the PIN detector has made it possible to determine the structure of the detector, which has been fundamental to know with accuracy the energy deposited in the dead layers and in the bulk of the detectors. This information is crucial to calculate, for each angle, how many and where carriers have formed along the ion trajectory. Angle-dependent LGAD experiments (up to 50°) have established how the gain increases with decreasing λ-parameter, while measurements for larger angles (up to 85°) have resulted in a change of trend in the gain behavior upon entering the microplasma formation regime, as from a critical angle of 57° the Bragg peak is deposited within the active bulk. One advantage of the IBIC technique proposed in this study over the use of TCT-lasers or Sr-90 sources is its better spatial resolution. Furthermore, in comparison with the previous work [12], performed with a collimated Sr-90 source and where the maximum angle of rotation was limited to 14°, in our study we used a monoenergetic and focused ion beam, which allows to define more precisely the angle of incidence and to increase it up to 90° by rotating the sample in vacuum.

## 2. Experimental Details

### 2.1. LGADs

The samples studied in this work are a PIN and a LGAD detector manufactured by the Centro Nacional de Microelectrónica (IMB-CNM-CSIC) [14]. Both detectors come from the same wafer and underwent the same fabrication process and are identical except for the *p*^+^ implant (gain layer) in the case of the LGAD. The samples were fabricated on a 347 µm thick Si-on-Si wafer, where a 50 µm thick high-resistivity <100> FZ wafer is bonded to a low-resistivity, 300 µm thick, <100> Czochralski wafer. The boron dose implanted, with an energy of 100 keV, to create the multiplication layer was 1.5 × 10^13^ at/cm^2^.

**Figure 3:**
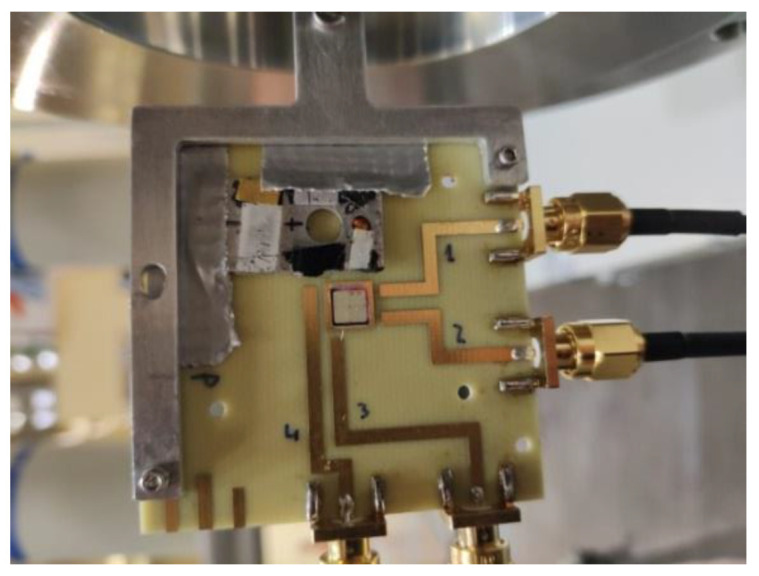
LGAD mounted in a dedicated PCB for IBIC, TRIBIC and TCT measurements installed in the sample holder of the CNA nuclear microprobe. On top of the detector there is an aluminum plate with scintillator materials, quartz and a copper grid for beam localization and focusing.

**Figure 4:**
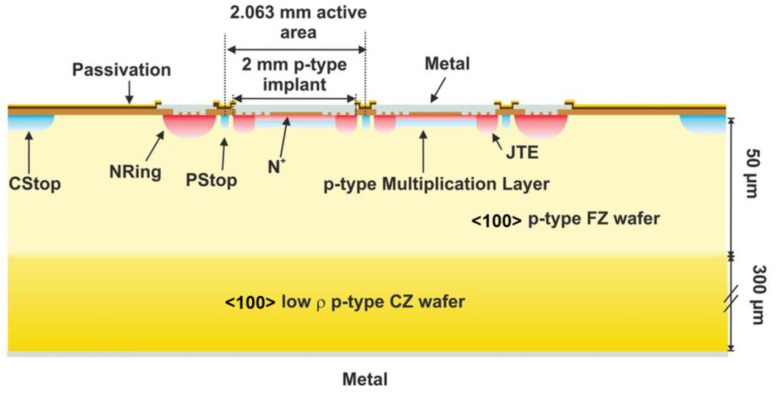
Schematic cross section of the LGAD of 2 × 2 pixels from the CNM production run 10478.

The samples consist of a matrix of 2 × 2 pixel detectors with an area of 2.063 × 2.063 mm^2^ each. The four pixels of the matrix were connected via bond wires to four independent 50 Ω vias ending each one on an SMA connector used to output the signal and to bias the detector. The PCB was designed to be compatible with the TCT system at the CERN Solid State Detector (SSD) laboratory and the nuclear microprobe at the Centro Nacional de Aceleradores (CNA), where the IBIC measurements were carried out. Figure 3 shows one of the detectors mounted on the PCB and placed in the sample holder of the CNA nuclear microprobe. A cross section of the LGAD diode is shown in Figure 4.

**Figure 5:**
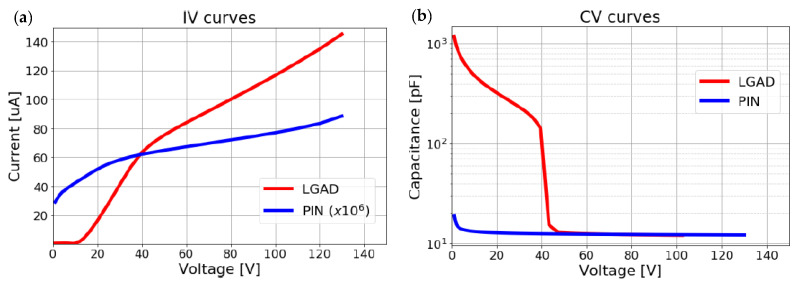
Electrical characterization of PIN and LGAD detectors: (**a**) I-V curves; (**b**) C-V curves.

The electrical characterization (I-V, C-V) of the detectors was performed at the SSD lab at CERN. From the results, shown in Figure 5, the LGAD main parameters were extracted, these are: *V*_*gl*_~38 V, *V*_*fd*_~43 V, *V*_*bd*_~140 V (*V*_*gl*_: Gain layer depletion voltage, *V*_*fd*_: Full depletion voltage, *V*_*bd*_: Breakdown voltage). The results shown in this paper correspond to a single pixel.

### 2.2. Experimental Set-Up

**Figure 6:**
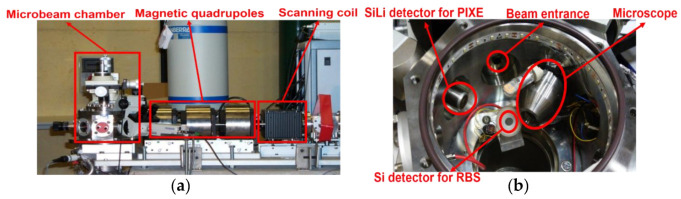
(**a**) Main elements of the microbeam system at CNA. (**b**) Zenithal image of the vacuum chamber into which the samples are inserted.

The IBIC and TRIBIC measurements were performed at the nuclear microprobe beamline of the CNA 3 MV tandem accelerator (Figure 6) [15]. The beam passes through a scanning coil which is the responsible of deflecting the ion beam to scan areas of a defined size. The standard OM-25 scanning coils are designed to operate with a quadrupole focusing system and MeV energy ions. The OM-150 coupled triplet is the element responsible of focusing the ion beam. It consists of a coupled triplet of OM-50 high precision magnetic quadrupole lenses in Convergent-Divergent-Convergent (CDC) configuration. At the end of the line is the OM-70 sample chamber. It is compatible with high vacuum. Inside the chamber, there is an X-ray SiLi detector, a Si detector for charged particles measurements and a microscope for sample and beam observation. The TCT with infrared laser (IR) results were obtained at the SSD of the EP-DT group at CERN by the Instituto de Física de Cantabria (IFCA) Particle Physics and Instrumentation (PP&I) group. Detailed information on this set-up can be found in [12]. 

The fundamentals of both techniques are similar: a probe (laser pulse in TCT and protons, or another ion, in IBIC) generates an ionization trace through the detector and the carriers created in the active volume of the sensor move towards the electrodes by the effect of the electric field, inducing a current pulse that is processed by the electronic chain. Note that, in both cases, the total charge generated within the detector will be proportional to the energy deposited in it, the proportionality factor being the product g×1/ε×q, where g is the gain of the detector, ε=3.62 eV is the mean energy required to produce an electron-hole pair in silicon and $q=1.6\times 10^{-19}\;C$ is the elemental charge. In the case of the TRIBIC technique, the current signal induced in the electrodes is brought to the input of an oscilloscope either directly or after passing through a current amplifier with a high bandwidth so as not to change the shape of the signal. Thus, we can measure the temporal evolution of the induced signal, not only the total charge generated as in the IBIC technique.

The IBIC and TRIBIC studies were accomplished with a 3 MeV proton beam with count rate of a few tens of particles per second and performing a scan of 100 × 100 µm^2^ to avoid damage to the detectors during the measurements. For IBIC, the signal height was recorded as a function of the applied reverse bias voltage using a Canberra 2003BT preamplifier, a Tennelec TC245 amplifier with a shaping time of 1 μs and the OMDAQ ADC/MCA system from Oxford Microbeams [16]. The TRIBIC experiments were performed by connecting the detector signal directly to a Cividec C2 current amplifier (2 GHz, 40 dB) and the output of this amplifier to the 50 Ω input of a high bandwidth oscilloscope TeledyneLecroy HDO9404 (4 GHz, 40 GS/s), where 1000 signals were recorded and averaged to improve the signal-to-noise ratio. 

For angular measurements, a special sample holder was used which allows the sensor to be rotated completely in vacuum, i.e., from 0° to 360°, with an accuracy of 1°. Results up to 85° will be presented. All measurements in this work have been made at room temperature (~20 °C).

## 3. Results and Discussion

### 3.1. Detector Homogeneity and Structure

**Figure 7:**
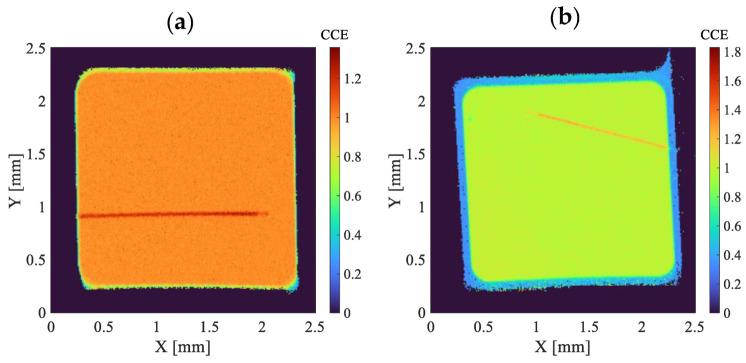
Weighted mean CCE maps (normalized to 1) for the PIN (**a**) and LGAD (**b**).

Before proceeding with the angular measurements, a study of the homogeneity of the Charge Collection Efficiency (CCE) along the surface of the detectors was performed. This preliminary step is necessary because, although the area scanned by the beam in the gain measurements (100 × 100 μm^2^) is small compared to the dimensions of the detectors (2 × 2 mm^2^), when working with grazing angles the beam projection in one direction is somewhat larger than 1 mm. For homogeneity measurements the beam was focused to a size of 3 μm and a 2.5 × 2.5 mm^2^ scan was performed covering the entire surface of the detectors. Figure 7 shows the weighted mean CCE maps (normalized to 1) for PIN (left) and LGAD (right) obtained with the detectors biased to 43 V.

Quantitative results indicate that the homogeneity of the CCE is better than 2% in the PIN and 4% in the LGAD. The line crossing both detectors correspond to the electrical bonding, since the protons passing through it reach the detector with a lower energy and, therefore, create a higher number of carriers in the active volume (CCE > 1) due to the increase of the stopping power at lower energies. In the case of the LGAD, a drop in the CCE is also observed at the periphery of the detector, which corresponds to the end of the multiplication layer.

Although the structure of the detectors is perfectly determined from the manufacturing processes, the thicknesses of the various passivation and metallization layers as well as the thickness of the active zone are not known with sufficient precision. This data is however necessary for a correct interpretation of the results obtained with the LGAD. To accurately determine the structure of the detector, a series of IBIC measurements at different angles of incidence were carried out on the PIN detector, which is identical to the LGAD except for the multiplication layer.

**Figure 8:**
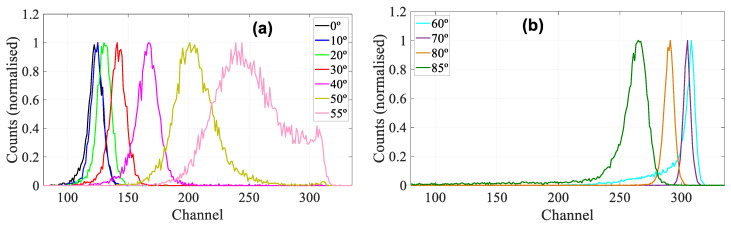
IBIC spectra from PIN diode in counts vs channel. (**a**): measurements up to 55° from which the thickness of the active layer is obtained. (**b**): the spectra for 60°, 70°, 80° and 85° from which the thickness of the dead layers (Al equivalent) is extracted.

Figure 8 shows the energy spectra (in normalized counts versus channel) obtained at different angles. On Figure 8a are the spectra measured up to 55°, where, for these conditions, all protons pass completely through the detector. As it can be seen, as the angle is increased, the peaks move towards higher channels, i.e., higher energies, which is due to a higher energy deposition in the detector due to the longer trajectory of the proton inside the sensor’s active volume. The spectra also become wider for larger angles due to the increased energy straggling. From the displacement of these peaks, the thickness of the active zone of the detector was obtained. Figure 8b shows the spectra for larger angles, between 60–85°. Under these conditions, all the protons stop in the sensitive volume of the detector, so the straggling decreases and at 70° we have a much thinner peak than at 50°, but the peaks broaden again when the energy lost in the dead layers of the detector becomes non-negligible, causing the peaks at 80° and 85° broaden again. In addition, as more energy is deposited in the dead layers, the energy deposited in the active volume is less, so these peaks shift to lower channels i.e., lower energies. In this case, the displacement of these peaks was used to determine the thickness of the dead layers of the detector, which for simplicity we have considered as an equivalent thickness of the Al electrode. 

**Figure 9:**
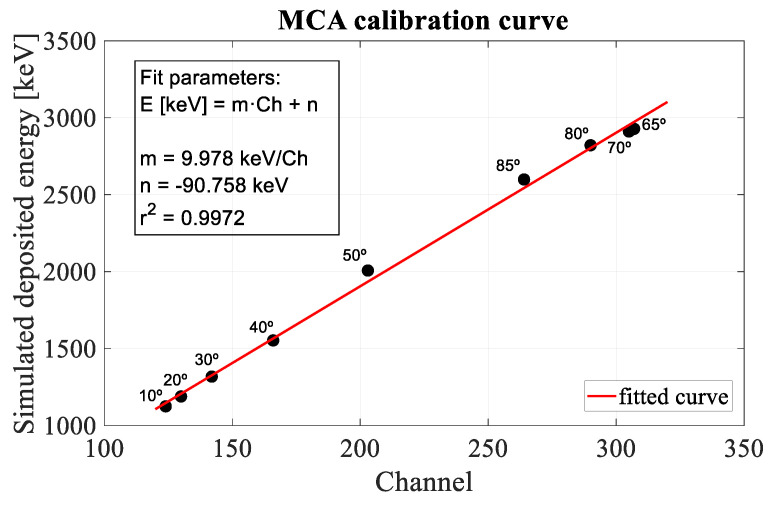
MCA calibration curve obtained by plotting the most probable value of the deposited energies at different angles simulated by the SRIM 2013 code against the experimental channels and calculating the line of best fit.

Using SRIM2013 software [17], simulations of energy loss in the Al and Si layers have been performed for all angles of incidence. The energy loss in the other two passive layers, the oxide and passivation, will translate into an overestimation of the thickness of the Al layer, keeping the energy deposition (and thus, charge) and thickness estimation for the silicon active volume still valid. By means of an iterative process, the thicknesses of both layers have been adjusted to obtain the best fitting calibration line for all experimental data (simulated deposited energy in Si vs centroid of the peaks). The values obtained, 1.5 µm Al and 48 µm Si, agree with those supplied by the IMB-CNM group (1.5 µm Al and 44 µm active volume + 4 µm multiplication layer [14]). Through this iterative process, the calibration curve of the Multichannel Analyzer was also found (as shown in Figure 9). 

### 3.2. Absolute Gain Curves in the Microplasma-Free Regime

Absolute gain curves were obtained by measuring the LGAD and PIN detectors under the same conditions, so that all parameters that vary with beam incidence angle, such as energy deposition in the sensitive volume, cancel out. The absolute gain was calculated as the ratio between the energy measured with the LGAD and the energy measured with the PIN detector, which is equivalent to the ratio between the charges collected at each of the detectors. 

**Figure 10:**
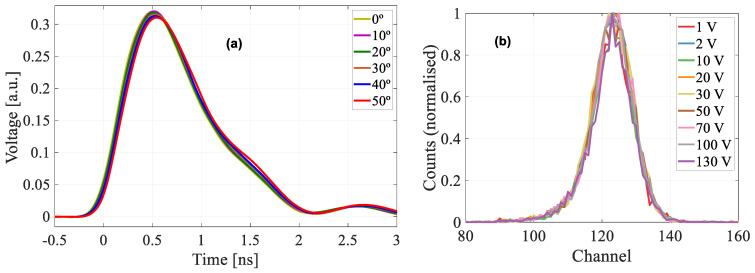
PIN measurements. (**a**) TRIBIC waveforms normalised to total deposited charge from 0° to 50°. (**b**) IBIC spectra in the bias voltage range 10–130 V.

The TRIBIC waveforms obtained from the PIN detector from 0° to 50° (Figure 10a) also served to ensure that, despite the increase in deposited energy with angle, in this range of values there is no formation of microplasmas in the detector volume, since otherwise the current pulse shapes would exhibit a slow component in their rise time attributed to the dispersal of the plasma [18]. Furthermore, the IBIC measurement at normal incidence of the PIN diode for all applied bias voltages up to 130 V (Figure 10b) showed no displacement of the peak, i.e., the collected charge was the same, which indicates that there are no diffusion effects due to the charge deposited on the electro neutral substrate under the active layer, so that the effects observed in the gain curves, must be related to the quenching of impact ionization due only to changes in the linear ionization density. 

**Figure 11:**
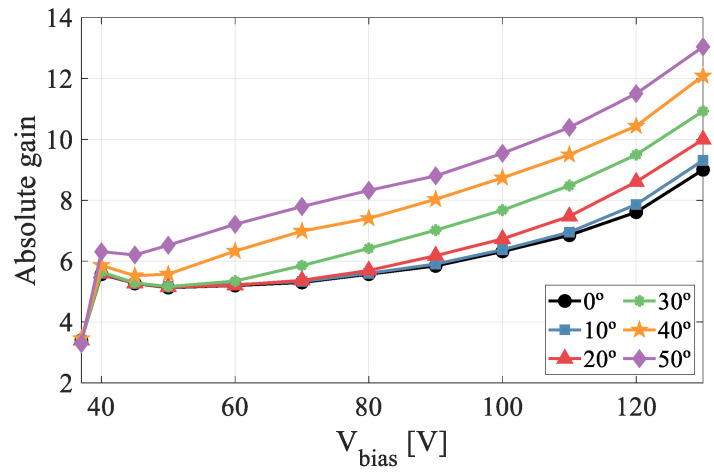
Absolute gain curves for angles of incidence from 0° to 50°.

The absolute gain results obtained in this microplasma-free regime are shown in Figure 11. The curve exhibits the lowest gain values for normal incidence (0°) and rises progressively as the angle of incidence increases until reaching the maximum value at 50°.

**Figure 12:**
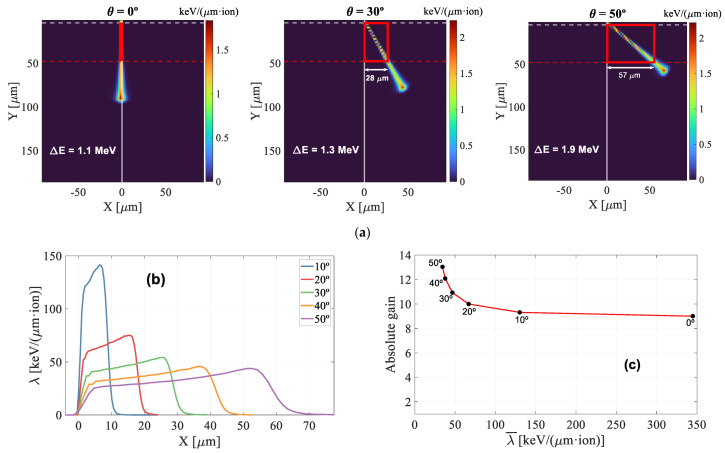
(**a**) SRIM simulations of the energy deposited by a 3 MeV proton beam entering from the top with an incident angle of 0°, 30° and 50°. The red dashed line indicates the end of the active layer. (**b**) Profile of the ionization density projected on the x axis for angles up to 50°. (**c**) Absolute gain versus the mean linear ionisation density at 130 V.

The SRIM simulations depicted in Figure 12a demonstrate that, although the energy deposited by the proton beam (ΔE) increases with angle, the projection of the ionization trace onto the multiplication layer grows much larger, making the linear ionization density a decreasing function with angle. It is important to note that since the ionization profile is depth-dependent, the λ-parameter will have different values along the X-axis projection, as shown in the SRIM simulation in Figure 12b. The mean value of the λ-profile (mean linear ionisation density) versus the absolute gain is plotted in Figure 12c. At 130 V, the absolute gain increases by approximately 45% when the detector is rotated by 50°.

**Figure 13:**
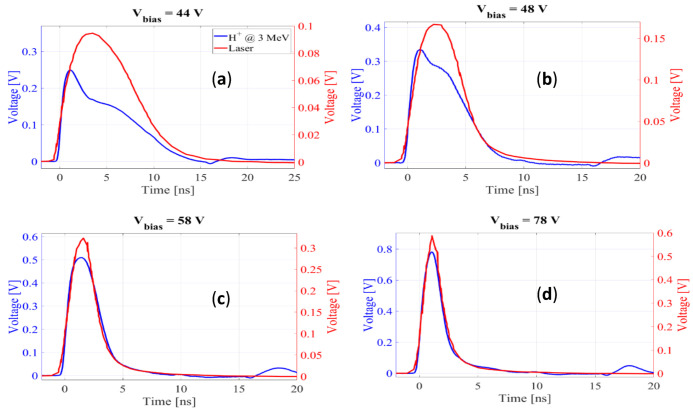
Comparison of TRIBIC and TCT-laser waveforms at different voltages at normal incidence (0°). (**a**) 44 V; (**b**) 48 V; (**c**) 58 V and (**d**) 78 V.

**Figure 14:**
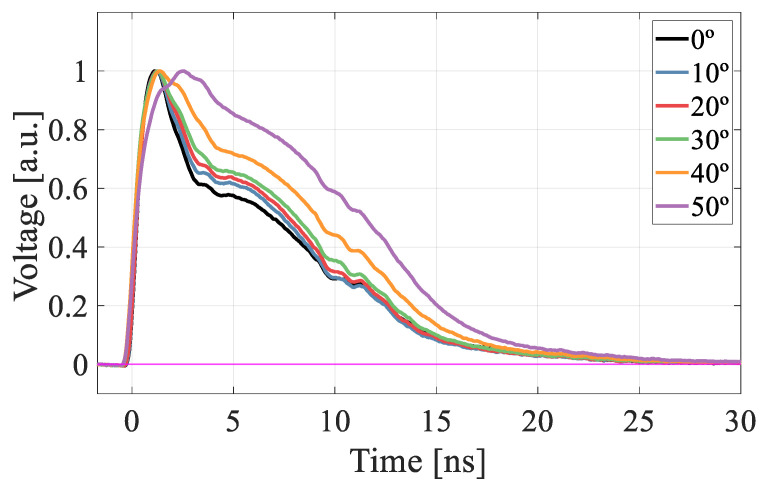
TRIBIC waveforms measured at 45 V for incidence angles from 0° to 50°.

To better understand the gain suppression mechanism as λ increases, we carried out a detailed study of the current-pulse shapes. In Figure 13, the TRIBIC waveform at normal incidence (0°) is compared with that obtained by TCT-laser. The analysis of the waveforms in their various components [19] indicates that the rapid rise is basically due to the contribution of the primary carriers and secondary electrons while the slower fall is mainly due to the movement of the secondary holes. At high voltages (Figure 13c,d) the waveforms become very short and lose much of its structure, so both techniques give practically the same signal. However, for voltages near the onset value (Figure 13a,b), although the TRIBIC and TCT-laser signals have a similar rise time and duration, their shape is very different, with the proton-induced signal presenting a clear deficit in the secondary holes component. This difference appears as a consequence of the gain suppression observed in Figure 2, therefore, the secondary holes component would be expected to become higher as the gain increases. This behavior is indeed the one observed in the TRIBIC measurements carried out as a function of the angle for a voltage of 45 V, so that the temporal structure can be resolved (Figure 14), where, as the angle of incidence increases up to 50° (and hence the gain) so does the relative contribution of the secondary holes.

### 3.3. Absolute Gain Curves in the Microplasma Regime

Studies of gain suppression in the absence of microplasmas are very scarce, in fact as far as we know there is only the parallel research carried out by the group at CERN’s SSD laboratory [12] and this work. On the other side, research on the quenching of impact ionization or the formation of plasmas in Si detectors are more numerous [11,18,20,21]. However, in these previous works, a high carrier injection was used, either by using the Two Photon Absorption (TPA) technique or by irradiating the devices with heavy ions such as He, C, O and N. In this paper, to study gain suppression in the plasma formation regime with 3 MeV protons, the rotation angle has been increased to get the Bragg Peak inside the bulk of the LGAD detector, since at that point the injected carrier density generates a micro-volume of ionization similar to the use of TPA.

**Figure 15:**
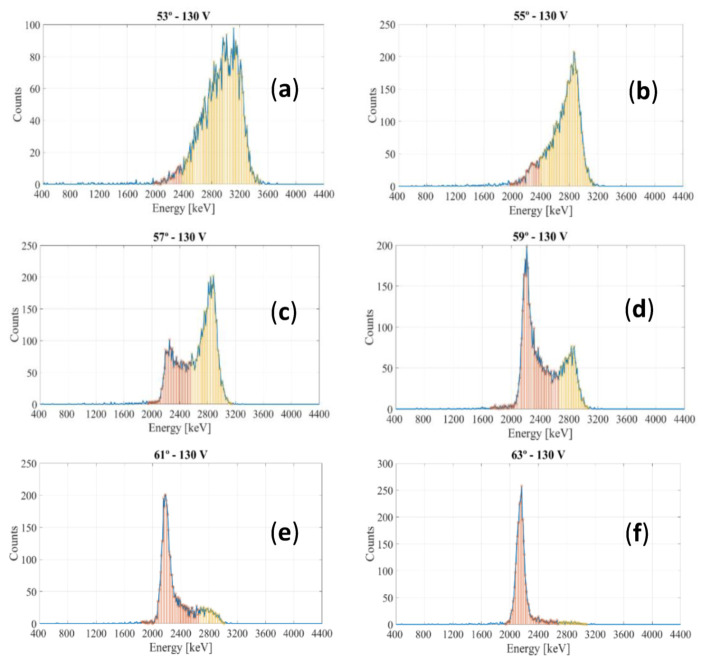
IBIC measurements at 130 V around the critical angle. (**a**) 53°; (**b**) 55°; (**c**) 57°; (**d**) 59°; (**e**) 61° and (**f**) 63°. The number of protons that stop within the active volume and the number of protons that are transmitted are represented in red and yellow colors, respectively.

To find the critical angle at which the Bragg Peak falls into the detector, IBIC measurements were performed by changing the angle of incidence in steps of 2° around the value found by SRIM for which there were no transmitted protons (at 64° incidence, only 1% of protons are transmitted). The obtained spectra are shown in Figure 15. At 53°, practically all protons pass through the detector and the spectrum shows a broad peak with a distribution of pulses mostly around 3042 keV. However, this distribution changes and two peaks start to appear as the angle increases. The peak at higher energies corresponds to the transmitted protonswhile the peak at lower energies is that of the protons stopping inside the active zone of the detector, as proven further on by the transmission curve. From this result, it becomes evident how the gain decreases visibly when the particles leave their full energy in the detector. Moreover, within a given angular range two different gains coexist, although with different probability, when one part of the ions passes through the active thickness while the other does not. At 63°, practically all the protons stop in the detector (leaving their full energy behind) so that the peak not only appears at lower energy but also becomes narrower since the straggling decreases as all the protons deposit the same energy and the effect of the statistical fluctuation in the deposited energy disappears.

**Figure 16:**
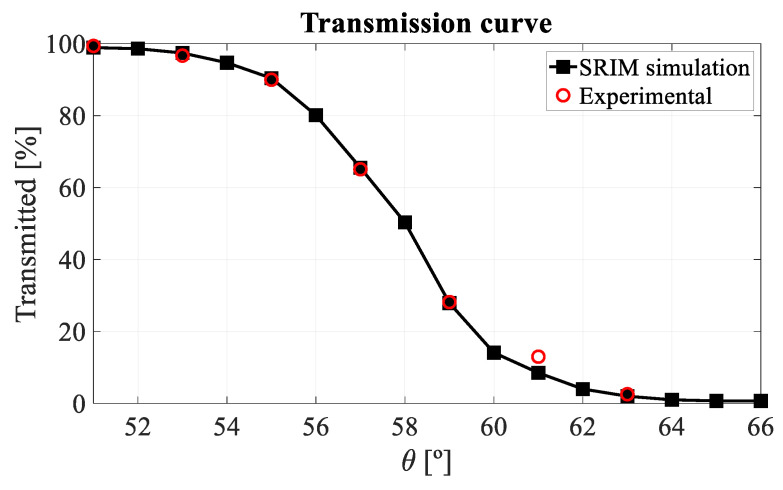
Experimental and theoretical (obtained with SRIM2013) transmission curves.

To confirm that the interpretation of the two peaks appearing in the IBIC spectra is correct, a transmission curve has been generated as a function of the angle by calculating the percentage of transmitted protons as the number of counts in the high energy peak divided by the total number of counts in the spectrum. This curve has been compared with the theoretical one, obtained by simulation with SRIM2013 (Figure 16).

**Figure 17:**
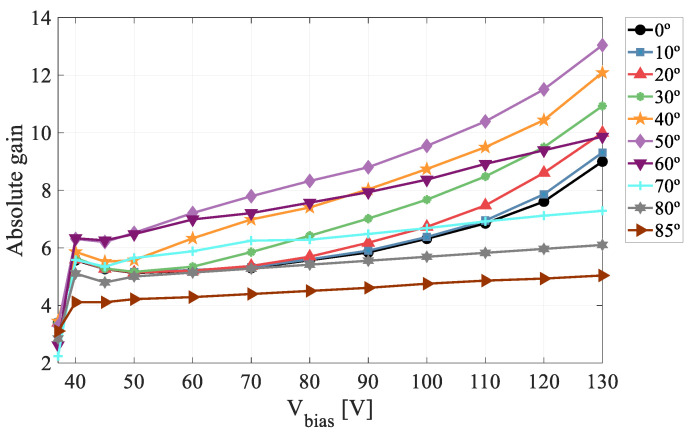
Absolute gain curves for angles from 0° to 85°.

**Figure 18:**
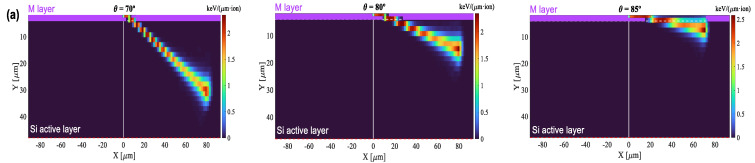

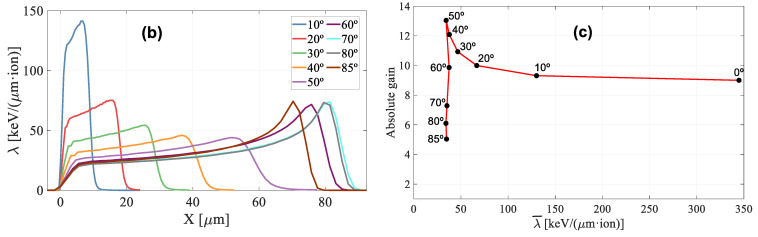
(**a**) SRIM simulations at 70°, 80° and 85°. Purple area is the multiplication layer. (**b**): Ionization density projected on the gain layer curves up to 85°. (**c**): absolute gain versus mean linear ionisation density at 130 V.

Figure 17 shows all the gain curves obtained in this work (for angles between 0° and 85°). After reaching its maximum value at 50°, the gain shows a decreasing behavior from 60° on, with gain suppression for 80° and 85° being even higher than for normal incidence. Moreover, for large angles, a lower dependence on the applied voltage is observed. To determine whether this drop in the gain could be explained, as in the previous section, by changes in the λ-parameter, the corresponding SRIM2013 simulations were performed (Figure 18). From 60° and above, the λ-profiles show relatively high values at the end of the range (similar to those calculated for 20°) so that the gain is expected to decrease with respect to that measured at 50°. On the other hand, it is clear that the sharp drop in gain between 60° and 85° cannot be attributed to variations in λ because both the profile and mean value remain almost constant (Figure 18b,c). We believe that the behavior of the LGAD for large incidence angles is closely linked to the fact that, from 60° on, the Bragg peak falls within the sensitive volume of the detector. For these conditions, the density of electron-hole pairs is high enough to form a plasma-like cloud of charge that shields the inside of the track from the influence of the electric field. During the plasma time, i.e., the time required for the plasma cloud to disperse to the point where normal charge collections proceeds, the carriers in the interior of the track do not drift although they may move by diffusion. This ambipolar diffusion produces a movement of carriers in all directions [21], causing the charge density to decrease. This phenomenon is however less effective when the Bragg peak is deposited closer to the multiplication layer because the carriers have less time to diffuse before being collected, so that, for the same initial value of λ, the gain should decrease at a higher angle of incidence. Another important aspect to keep in mind is that the multiplication factor (gain) grows exponentially with the distance traveled by the electrons in the multiplication layer [22]. While electrons created in the substrate travel the entire multiplication layer before reaching the anode, those produced in this same layer will travel a shorter distance, resulting in a lower gain. This process becomes more relevant for grazing incidence, in agreement with our experimental results.

**Figure 19:**
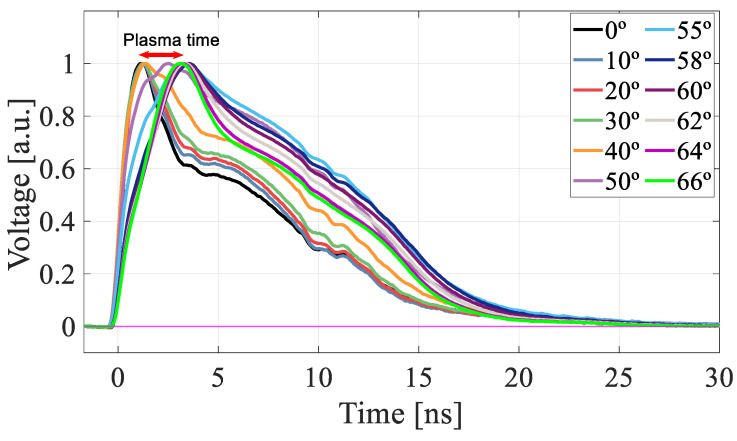
TRIBIC waveforms at 45 V for angles up to 66°.

As shown in Figure 19, the microplasma formation in the detector bulk is clearly visible in the time evolution of the current-pulse signals. Indeed, the TRIBIC experiments performed at 45 V for angles up to 66° confirm that when the Bragg peak falls inside the detector, the waveform rises more slowly, introducing a delay of about 3 ns (plasma time) due to the shielding of the carriers when the plasma cloud is formed. Finally, it is worth noting that the correlation discussed above between the relative component of secondary holes and gain is also observed in this regime of microplasma formation.

## 4. Conclusions

IBIC measurements using a rotating sample holder in a nuclear microprobe have allowed us to perform, for the first time, a detailed study of the effects of gain suppression in LGAD detectors for a broad range of incidence angles (between 0° and 85°). In the small angle regime, our results obtained with a 3 MeV proton beam are in qualitative agreement with those found using electrons from a Sr-90 source [12]. The gain curves up to 50° show a monotonic growth as the angle of incidence increases. This behavior correlates with a progressive decrease in the projection of the carrier’s density along the multiplication layer. This magnitude, together with temperature or applied voltage, is therefore an important factor to consider for a better understanding and control of the gain in these devices. In fact, the variations in gain caused by the passage of MIPs through the LGAD at different angles of incidence could lead to a larger dispersion in the values of temporal resolution for the same experiment. 

Time-resolved IBIC measurements carried out at voltages close to the onset value present a substantial deformation of the current-pulse shape due to a deficit in the secondary holes’ component, and this characteristic is accentuated when there is greater gain suppression. However, this behavior is not observed for nominal operating voltages as the signals become much shorter and lose much of their time structure.

For incidence angles such that the proton range approaches the end of the active layer of the LGAD, the IBIC spectra show a distinctive behavior, with the simultaneous appearance of two distinct peaks. Comparison with SRIM simulations indicates that the peak at high energy is caused by protons transmitted to the electroneutral substrate while the signal at lower energy corresponds to protons depositing their full energy on the detector. This demonstrates that when the Bragg peak falls within the sensitive volume of the detector the gain suppression is considerably enhanced. Moreover, the corresponding TRIBIC waveforms have longer rise times, indicative of a plasma formation along the ion track. Under these conditions, the trend of the gain curves is modified, showing a sharp decrease in the gain as the angle increases. This can be understood in terms of the ambipolar diffusion of the carriers and by the greater number of electrons created directly by the proton beam in the multiplication layer, which travel less distance to the anode and therefore have less ability to create secondary ionizations.

In a future work we plan to study in detail the effect of ambipolar diffusion and the creation of e-h pairs in the multiplication layer. To do this, the IMB will fabricate LGAD detectors surrounded by a very thin dead layer of silicon (about 5 microns instead of the 300 microns of current detectors) so that we can make lateral IBIC measurements (that is, with an incidence angle of 90°). By impinging with the proton beam on the active layer at different distances from the detector surface (from the electroneutral substrate to the multiplication layer) we will be able to vary the diffusion time of the carriers and, consequently, study the importance of this mechanism. In addition, the beam can be directly incident on the multiplier layer to analyze the gain degradation due to this factor alone.

## Data Availability

Not applicable.

## Abbreviations

Acronym	Definition
ADC	Analog-to-Digital Converter
APD	Avalanche Photo Diode
ATLAS	A Toroidal Large hadron collider ApparatuS
CCE	Charge Collection Efficiency
CERN	Conseil Européen pour la Recherche Nucléaire
CMS	Compact Muon Solenoid
CNA	Centro Nacional de Aceleradores
CNM	Centro Nacional de Microelectrónica
CSIC	Consejo Superior de Investigaciones Científicas
EP-DT	Experimental Physics—Detector Technologies
HGTD	High Granularity Time Detector
IBIC	Ion Beam Induced Current
IFCA	Instituto de Física de Cantabria
IMB	Instituto de Microelectrónica de Barcelona
IR	InfraRed
LGAD	Low Gain Avalanche Detector
LHC	Large Hadron Collider
MCA	MultiChannel Analyzer
MIP	Minimum Ionizing Particle
MTD	Minimum ionizing particle Timing Detector
PIN	p-type—Intrinsic—n-type
PP&I	Particle Physics and Instrumentation
SMA	SubMiniature version A
SSD	Solid State Detector
TCT	Transient Current Technique
TPA	Two Photon Absorption
TRIBIC	Time-Resolved Ion Beam Induced Current
US	Universidad de Sevilla
λ	Linear ionization density
